# Unraveling the enigma: elucidating the relationship between the physicochemical properties of aluminium-based adjuvants and their immunological mechanisms of action

**DOI:** 10.1186/s13223-018-0305-2

**Published:** 2018-11-07

**Authors:** Emma Shardlow, Matthew Mold, Christopher Exley

**Affiliations:** The Birchall Centre, Lennard Jones Laboratories, Keele University, Keele, Staffordshire ST5 5BG UK

## Abstract

Aluminium salts are by far the most commonly used adjuvants in vaccines. There are only two aluminium salts which are used in clinically-approved vaccines, Alhydrogel^®^ and AdjuPhos^®^, while the novel aluminium adjuvant used in Gardasil^®^ is a sulphated version of the latter. We have investigated the physicochemical properties of these two aluminium adjuvants and specifically in milieus approximating to both vaccine vehicles and the composition of injection sites. Additionally we have used a monocytic cell line to establish the relationship between their physicochemical properties and their internalisation and cytotoxicity. We emphasise that aluminium adjuvants used in clinically approved vaccines are chemically and biologically dissimilar with concomitantly potentially distinct roles in vaccine-related adverse events.

## Review

There are two aluminium based adjuvants (ABAs) commonly used in vaccines [1]. Alhydrogel^®^ is a semi-crystalline form of aluminium oxyhydroxide (AH) and AdjuPhos^®^ is an amorphous salt of aluminium hydroxyphosphate (AP). A sulphate salt of the latter (AAHS) is also listed as being one component of an adjuvant system used in vaccinations against HPV [2]. Alhydrogel^®^ and AdjuPhos^®^ are commonly referred to as clinically-approved ABAs [3] and yet this is not the case. There are no ABAs which have been approved for intramuscular or subcutaneous injection into humans. There are no requirements for their approval, they are only ‘approved’ as part of vaccine preparations.

Aluminium-adjuvanted vaccines have a long history of clinical successes and a commensurately long history of vaccine-related adverse events [4]. Since there is no requirement to demonstrate the safety of ABAs one could quickly surmise that adverse events following vaccination are the direct or indirect effects of ABAs. This is almost certainly true as the phenotypes of most adverse events are unrelated to the disease being treated and are reproduced, at least in the short term, in ‘clinical approval trials’ where ABAs are the ill thought through placebos [5]. It is almost certainly the widespread use of ABAs and/or other aluminium-adjuvanted vaccines as placebos in vaccine safety trials that have guaranteed clinical approval of many vaccines. The numbers of individuals affected by an adverse event is largely determined by how widely a vaccine is administered (essentially a percentage of those inoculated) while the clinical manifestation and severity of an adverse event may be almost bespoke to the vaccine recipient. To better appreciate how ABAs are responsible for adverse events we must start with an understanding of their physicochemical properties within the physiological environment of the injection site. Indeed such information might also be a starting point for the design of novel and safer ABAs for future applications in vaccines.

The clinical significance of the physicochemical properties of ABAs begins prior to their combination with the antigen of interest. This is because the nature of their interaction with antigen will be influenced by the surface they present for adsorption of antigen and such surfaces will depend upon the particle size profile of the adjuvant material as well as such aspects as surface functionality, e.g. hydroxyl or hydroxyphosphate, and the overall surface charge in the vehicle used to suspend them. The antigen, upon mixing, may then be strongly adsorbed to adjuvant surfaces or it may simply be occluded within aggregates of adjuvant. Since there will also be some dissolution of ABA at this point then there may also be direct interactions, such as ionic binding, between antigen and ${\text{Al}}^{ 3+ }_{{({\text{aq}})}}$. Even before its injection a vaccine preparation can be extremely heterogeneous in respect of the presentation of the adjuvant-antigen complex. The same preparation is then subject to its passage through a hypodermic needle and into the even more diverse environment of the injection site, whether it is intramuscular or sub-cutaneous.

One aspect of the injection of a vaccine that invariably seems to be ignored is the immediate acuity of such an exposure to aluminium. The concentration of total aluminium in the vaccine can be as much as 63 mM (1.7 mg/mL) and immediately upon its dilution into interstitial fluid its concentration in the immediate vicinity of the injection site will remain high, mM, for minutes if not hours following the injection. The solubility of the ABA will increase markedly upon its dilution at the injection site, both due to dilution but also the influence of myriad solubilising moieties reacting with ${\text{Al}}^{ 3+ }_{{({\text{aq}})}}$, and commensurate with this will be a significant increase in the biological availability of aluminium [6]. Toxicity will be inevitable under such conditions and might be expected to be manifested as necrotic cell death and a subsequent inflammatory response. Herein, perhaps, is the origin of the potent adjuvant activity of aluminium salts; an immediate potency (often manifested as a simple reddening and swelling of the skin at the site of the injection) which will depend upon the presentation of the aluminium salt in question and, critically, the physiological response of the recipient.

While some degree of toxicity and cell death is probably inevitable, all subsequent responses to the adjuvant will bear the signature of an individual and these iatrogenic effects can range from benign to fatal. In every case, we might expect some degree of innate immune response to the antigen and that this will involve harvesting of adjuvant/antigen complexes by infiltrating immune-responsive phagocytic cell types. We know that ABAs are taken up by monocytic T helper 1 (THP-1) [7] cells and we also know that these cells respond differently to different ABAs. What we do not know is how such different responses are manifested in the innate response. For example, is endocytosis of ABA a prerequisite for the successful generation of innate immunity to the associated antigen? The apparent capability for monocytes to endocytose significant amounts of ABA without suffering obvious toxicity adds the additional dimension of these cells being vehicles for the trafficking of aluminium adjuvant throughout the body and into the brain [8].

Different ABAs are neither physically nor chemically equivalent and importantly ABAs are significantly dissimilar to other non-ABAs such as silica or uric acid. It is a mistake to make the assumption that adjuvants share an exact or similar action. It is, therefore, similarly mistaken to assume that all adjuvants are capable of adverse events following vaccinations.

## The physicochemical properties of ABAs and their respective contributions to the immunological potency of vaccines

The physicochemical characterisation of ABAs remained in its infancy until 1990, when many studies appeared on the subject in an attempt to address immunological issues resulting from inconsistent batch production. Indeed, the lack of industrial standardisation and optimisation involved in vaccine formulation was widely regarded as the reason behind the fluctuating efficacy of preparations containing identical base components. The culmination of these efforts was the identification of several physicochemical properties that influenced the adjuvanticity of aluminium salts, namely hydrated colloidal structure, particle size and surface charge [1]. While some attempt has been made to investigate the correlation between adjuvant physicochemistry and immunopotentiation (see [9, 10] for past reviews on the subject), the nuances of this relationship remain relatively enigmatic. This section will explore the correlation between these two parameters based on an evaluation of current literature and ultimately identify which fundamental issues remain to be addressed.

### The structural characteristics of ABAs and their role in the immunological response towards ABAs

#### Aluminium hydroxide gels

The aluminium hydroxide salts used in vaccines are more structurally complex than inferred by their generic chemical designation. The structure of contemporary aluminium hydroxide adjuvants resembles that of boehmite (AH) rather than that of the polymorphic hydroxide gels synthesised in 1958 [11]. Alhydrogel^®^, for example, has infrared (IR) stretching and bending vibrations located in almost identical positions to those obtained specifically for AH, which include a strong hydroxyl deformation band and stretching shoulder around 1070 and 3100 cm^−1^ respectively [12]. However, as noted by Wang et al. the additional hydroxyl deformation peaks at 835, 890 and 965 cm^−1^ imply that the structure of these adjuvants is significantly more hydrated than that of well-ordered boehmites [13], an observation which may be attributed to the specific conditions under which they are synthesised. Utilising lower temperatures and more acidic conditions during the synthesis of boehmites encourages the formation of small crystallites containing an abundance of non-stoichiometric water molecules, which are present both at the surface interface and intercalated between octahedral layers [14, 15]. The resulting crystallites are fibrillar in morphology expressing preferential elongation in the direction of the c axis and are in the region of 4.5 nm × 2.2 nm × 10 nm in size [13, 16–18], which is diminutive when compared to the larger plate like structures observed for highly crystalline boehmites [14]. These structures are thus characteristically more disordered and produce characteristic Bragg reflections which are broad in nature and diminished in intensity [14]. As a consequence, these poorly crystalline boehmites (PCB) have a substantially larger surface area than that of highly crystalline aluminium hydroxides such as I-gibbsite (510 m^2^/g [18] vs. 29 m^2^/g [19]). It is should be noted, however, that the value of the former is significantly reduced during the process of vaccine formulation. Immersion within physiological saline (0.15 M NaCl), for example, prompted a decrease in the specific surface area of two commercial AH adjuvants (Alhydrogel^®^ and Rehydragel^®^) from 350 and 300 m^2^/g to 225 and 150 m^2^/g respectively [20].

From a vaccinological perspective, the larger relative surface area of PCB gels appears to offer significant advantages in terms of antigenic adsorption over more crystalline analogues or those composed primarily of highly ordered polymorphic hydroxides. The AH adjuvants used in clinical vaccines, for instance, are touted as having high adsorptive capacities for myriad proteins under isotonic conditions. Notable examples include the adsorptive capacity of Alhydrogel^®^ for bovine serum albumin (BSA), β-casein and hepatitis B surface antigen (HBsAg) in 0.15 M NaCl, all of which are substantial at 1.25, 3.43 and 1.43 mg/mg Al respectively [21]. Despite these considerable values, the quantity of adjuvant used within prophylactic vaccines often greatly exceeds that of the antigen and thus ensures that complete adsorption is consistently achieved. These events induce conformational changes in the structure of the bound antigen, which theoretically increases both their proteolytic susceptibility and subsequent presentation to T cells via MHC class II molecules [22]. Furthermore, maximal antigen adsorption is reportedly necessary for the efficacy of immunisations containing *S. pneumoniae* amongst others [23]. The relationship between adsorptive capacity and immunopotentiation, however, remains equivocal and facilitating a higher percentage of antigenic adsorption within the vaccine preparation itself does not guarantee that the formulation will exhibit superior immunogenicity (see [24] for an extensive review of this subject). It is thus reasonable to conclude that the dependence of vaccine potency on adjuvant-antigen interaction is component specific and as such should be evaluated on an individual formulation basis.

The structural order of AH is also an important consideration in terms of tailoring their specific adjuvant activity when administered as part of a vaccine. Synthetic boehmite nanorods of lower structural order have been shown to stimulate the production of significantly elevated levels of IL-1β and IL-6 in vivo in comparison to their more crystalline counterparts [25]. The presence of these pro-inflammatory cytokines is indicative of the classical Th2 polarisation typically associated with aluminium-adjuvanted vaccines [26–31]. Despite these initial results, the IgG1 titres potentiated by nanorod analogues were visually comparable 2 weeks following immunisation (fibrous vs. crystalline rod—*ca* 1 ± 0.4 and 0.6 ± 0.4 ng/mL respectively), although it should be noted that statistical comparisons between candidates were not explicitly made or referred to within the text. Conversely, however, studies using more amorphous nanorods demonstrated that the level of IgG1 potentiated by these materials was significantly greater than those elicited by Alhydrogel^®^ 61 days post vaccination [29]. Nevertheless, this apparent disconnect between the initial innate and adaptive immunological responses noted in the former is consistent with the well-established belief that IL-6, in particular, has little to no influence upon the enhancement of adjuvant-associated immunoglobulin levels in vivo [30]. Similarly, more recent research has demonstrated that while IL-1β appeared to promote the recruitment of leukocytes towards the injection site [31], its abolition also had a negligible impact upon the generation of murine OVA specific IgG [32–36]. While many studies, including this one [25], argue that the adjuvanticity of aluminium salts is mediated by the formation of the inflammasome complex [37, 38], there is now mounting evidence to suggest that the magnitude of antibody titres generated at early junctures post vaccination may be somewhat independent of these innate signalling pathways and structural crystallinity. Furthermore, morphological differences in the structure of these adjuvants may be more relevant in this context as rod-like candidates generated higher overall antibody titres than platy or polyhedron type structures.

Allergic IgE responses were also diminished in mice vaccinated with more crystalline derivatives [25]. The reduction in IgE levels as a consequence of a departure from the application of more fibrous morphologies could theoretically be attributed to a reduction in IL-18 production, another well documented component of the PCB cytokine milieu [39]. This cytokine, in conjunction with other members of the IL-1 superfamily, can also drive Th17 differentiation [40], an immunological pathway activated in mice immunised with adjuvanted vaccines containing intermediate to high doses of whole-cell bacterial antigens [41]. Furthermore, IL-18 has been shown to positively influence the production of both mature and precursor forms of IL-1β [42], which is consistent with the attenuation of the latter in mice challenged with materials of higher crystallinity. It is thus evident that the solid-state chemistry of AH adjuvants may play a more significant role with regards to the regulation of immunoglobulin class switching. Explicit modulation of IgE responses via this route could be used to temper the increasing incidence of local hypersensitivity reactions in paediatric populations vaccinated with PCB containing vaccines (see communication by Bergfors et al. for a short summary of this topic [43]). If carefully conceived and implemented, it is feasible that such augmentations could improve the safety of such formulations without compromising their immunological efficacy.

Exposure to these nanorods also induced the secretion of significant levels of IL-12 from BDMCs after 8 h, which putatively suggests some degree of preferential polarisation towards a cell-mediated response [25]. It is thus tempting to speculate that the presence of IL-12 may drive some degree of IFN-γ dependent Th1 activation in this case. Though relatively uncommon, incidences of such phenomena have been observed following the administration of prophylactic formulations adjuvanted with commercial equivalents [44–47]. Indeed, novel proteomic approaches have highlighted a significant role for IFN-γ signalling pathways in the immunological mechanisms potentiated by AH, which specifically included increased expression of HLA molecules (class I and II) and the generation of CD8^+^ memory cells [48]. The ability of PCB containing vaccines to enhance the generation of the latter is considered of particular importance in relation to the acquisition of sustained immunity towards pathogenic challenge [46]. Aluminium salts are, however, notoriously inept at inducing the terminal differentiation required to promote robust cell-mediated responses [34, 46, 49]. In conjunction, this behaviour implies that sustained IL-12 production does not feature prominently in the immunological response towards commercial adjuvants [50]. In fact, others have demonstrated that formulations containing Alhydrogel^®^ actively inhibited APC derived IL-12 in a predominantly dose-dependent manner both alone and in the presence of a CpG oligonucleotide antigen [51]. The discrepancies between the IL-12 responses generated in reaction to synthetic vs. commercial boehmites in this case may lie in the different sampling durations chosen by each group (8 vs. 24 h). Additionally, while IL-12 signalling is indeed crucial with regards to the activation of Th1 driven responses [52], it is also capable of evoking B cell-mediated humoral immunity independently of IFN-γ by acting as an initial primer for the production of IL-6 and IL-10, thereby inhibiting and self-regulating its own biological activity [53–55]. It is possible, therefore, that early IL-12 production by dendritic cells (DCs) in response to vaccines adjuvanted with AH may be indirectly responsible for directing murine immune responses towards a Th2 profile.

As a final remark regarding the study by Sun et al. [25], the authors also purport to identify several analogues that out-performed a “commercial” adjuvant competitor Imject^®^ alum. It is worth noting however, that Imject^®^ alum was a poor comparative choice in this case as it is neither used in clinical vaccines nor is it an AH adjuvant [56]. In fact, this suspension is known for its poor immunogenicity in comparison to clinically utilized AH gels [57] and its use in experiments designed to investigate the mechanisms surrounding ABA immunopotentiation has been openly criticised [10, 56]. The clinical relevance of these findings are therefore difficult to assess and emphasises that the choice of commercial materials in such experiments should be carefully considered in order for meaningful comparisons to be made.

#### Aluminium phosphate

Aluminium phosphate suspensions and their sulphated derivatives represent a secondary choice of adjuvant for inclusion within vaccines that require an inorganic immunopotentiator. When analysed via IR spectroscopy, commercial preparations exhibit functional characteristics synonymous with that of AP, including a strong phosphate peak at *ca* 1100 cm^−1^ and broad hydroxyl stretches in the range of *ca* 3500–3100 cm^−1^ [12]. These adjuvants lack translational periodicity by X-ray diffraction (XRD) [12] and due to their amorphous nature contain a high proportion of adsorbed water molecules at the surface interface (unpublished observations). By transmission electron microscopy (TEM), AP demonstrates an aggregated platy morphology composed of nanoparticles approximately 50 nm in size [12, 58]. Augmentations of synthetic conditions during the manufacture of this adjuvant have been shown to induce a shift in the ratio of surface hydroxyl: phosphate groups exhibited by individual batches [59]. The extent of molecular phosphate inclusion is particularly important as it influences the surface properties of adjuvant particulates and the resultant efficacy of immunological potentiation [60–63].

Given the information presented in the previous sections, one could reasonably predict that the amorphous nature of AP adjuvants could facilitate: (i) the increased reactivity of the adjuvant at the injection site and (ii) higher levels of serum IgE production. The former has been demonstrated to some degree using comparative studies in which AP was determined to be more cytotoxic than AH in vitro [64]. The latter is more difficult to justify as few studies directly compare allergic responses potentiated by AP and AH adjuvants or indeed crystalline vs. amorphous AP preparations. Nevertheless, the administration of AP adsorbed vaccines does appear to elicit both protracted and significant levels of IgE secretion in rodents [65]. However, IL-4 is an essential requirement for the induction of allergic response towards aluminium salts [28] and there is currently no evidence to suggest that IL-4 production is more pronounced when vaccines adjuvanted with AP are administered [44]. Furthermore, if another indicator is used, namely IL-18, the response of human PBMCs to AP was substantially reduced in comparison to that potentiated by AH [39].

The recent development of AAHS by Merck has prompted its preferential inclusion and consequent displacement of AP in many of its vaccine formulations. Characterisation of this adjuvant still remains in its preliminary stages and literature sources pertaining to such are increasingly uncommon. In lieu of XRD analysis, AAHS has been described as an amorphous mesh with a perhaps unsurprising morphological resemblance to AP [66, 67]. Due to the lack of afore-mentioned physicochemical studies, it is difficult to make objective comments upon the efficacy and safety of this preparation in relation to its structural characteristics. Furthermore, the only available comparative study demonstrated that while AAHS adjuvants elicited high levels of HPV specific IgG1 antibodies in mice immunized via the intramuscular route, these responses were not statistically different from those potentiated by AP [66]. Given the results of this study, the rationale for the use of AAHS over more traditional AP preparations is currently difficult to discern.

### The role of particle size in the immunological response towards ABAs

The substantial efficacy of particulate delivery systems with respect to both immunopotentiation and immunomodulation post vaccination is universally accepted [68]. Homogenous sub-micron or nano-particulate suspensions appear preferential with emphasis upon the use of the latter, a logic perpetuated by the inverse nature of the relationship between particle size and membrane permeability or cellular uptake [69]. The application of such a conclusion to the relative adjuvanticity of such populations in biological systems, however, is by no means unanimous.

The heterogeneity and aggregation capacity of ABAs in hydrodynamic systems, including those of the vaccine preparation and inoculation site, are persistent obstacles that hinder the investigation of the relationship between the relative size of these salts and the subsequent magnitude and pathway of immunopotentiation elicited in vivo. Many have relied upon the use of stable, monodisperse particulate models such as polylactide (PLA) in order to elucidate the importance of this association [70–72]. However, application of these trends to ABAs is spurious at best given the numerous physicochemical differences observed between these preparations and those containing aluminium.

Other approaches have sought to compare the immunological response to both micron and nano-sized aluminium particulates in order to minimise the variations in physicochemistry typically encountered when comparing ABAs. Perhaps unsurprisingly, AH nanoparticles and nanosticks out-performed their microparticle counterparts not only with regards to antibody generation but also induced a marked reduction in inflammation in situ [29, 73]. Contrary to numerous reports [7, 8, 64, 74–76], both reported virtually no cellular uptake of OVA-conjoined microparticles relative to the rapid internalisation of nanoparticles, an argument presented as a major contributor to their increased adjuvanticity along with that of enhanced protein adsorption. However, the increased cytotoxicity of such materials as evidenced by their ability to induce significant levels of uric acid production in vivo provides a more convincing explanation [77]. AH nanoparticles have also been able to potentiate potent CD8^+^ T cell responses, which makes them attractive potential candidates for vaccines against Tuberculosis [78] and those intended for immunotherapy [79]. While these studies are highly informative with regards to the development of more efficacious vaccine adjuvants, the application of their results with regards to elucidating the immunological mechanisms activated by clinically utilised ABAs is questionable. Moreover, the data presented in these studies challenges the well-established consensus that uptake of ABAs is a prerequisite for their immunological action, an increasingly improbable notion given the results of a recent study where aluminium adjuvanted vaccines were administered intramuscularly to rhesus macaques [80].

#### The particle size of ABA stock suspensions

Early characterisation of native ABA preparations by TEM identified that although composed of heterogeneous microparticles [81] the average size of both suspensions was < 10 µm [82]. Indeed, under conditions that promote maximum aggregation i.e. the point of zero charge (PZC), the average size of synthetic AP candidates peaked at *ca* 3 µm [58]. These attempts to ascertain the particle size distribution (PSD) of ABAs were nevertheless problematic and utilised methodology where analysis was conducted in an anhydrous environment. The removal of water at crystallite interfaces has been shown to facilitate the nucleation and subsequent growth of particulates and adjuvant-diluent interactions are a crucial mediator of systemic agglomeration in colloidal suspensions [83–88].

Hydrodynamic analyses of ABA suspensions also have their limitations mainly due to the polydispersity of the preparations themselves; however, such methodologies provide an informative reference PSD under relevant environmental conditions. Our group recently reported that the median particle size of Alhydrogel^®^ and Adju-Phos^®^ by dynamic light scattering (DLS) was *ca* 3 and 7 μm respectively [89]. The latter was in good agreement with that obtained by Kolade et al. using low angle laser light scattering for an equivalent commercial preparation (*ca* 6 μm) [90]. Others have used liquid particle counting techniques in order to demonstrate the diversity of micron sized agglomerates present within synthetic APs, which reportedly range from *ca* 2–100 μm [91]. Despite the smaller size values and monomodal distribution obtained via DLS, the high polydispersity of Alhydrogel^®^ solutions implied that several populations exceeded the maximum size limit of DLS. This was consistent with reports that described the PSD of this adjuvant as multimodal, with the contribution of additional peaks being recorded at *ca* 22 and 44 μm [92]. These results imply that the hydrodynamic size of ABAs is intricately complex. Furthermore, these hydrodynamic studies highlight that the elevated size of these colloidal suspensions may bear a distinct relation to their relative ability to form interactions with the aqueous environment through interfacial hydroxyl groups.

#### The particle size of ABAs within vaccine preparations

Adjuvanted vaccine formulations containing attenuated/inactivated pathogenic antigen associated with ABAs are usually administered in an isotonic solution of physiological saline. The matrix of this diluent is teaming with monovalent electrolytes which facilitate an increase in ionic strength relative to the minimalist environment of the native preparation.

When introduced into physiological saline, ABAs undergo extensive aggregation in comparison to that observed within ultrapure water (UPW) at equivalent concentrations of Al; however, a marked disaggregation is generally observed relative to that of the native stock [89]. The former can be attributed to a phenomenon known as charge screening, which actively reduces the repulsion between particulates [93]. The formation of smaller agglomerates in this case can be considered beneficial in terms of antigenic adsorption as preparations containing a higher proportion of larger entities typically exhibit lower proteinaceous absorption capacities [94]. Facilitating such events has been shown to increase the size of the resultant antigen-adjuvant complex [75, 89], which is likely to influence their subsequent recognition and uptake at the site of injection. Particulate coagulation has been traditionally considered to negatively impact the immunogenicity of immunisations in vivo; however, this remains largely dependent upon the extent of these events as preparations containing a smaller proportion of large agglomerates (> 10 μm) have been shown to be equally effective at potentiating humoral responses [83–87].

The influence of phosphate ions upon the PSD of ABAs has yet to be fully explored. Preliminary studies have suggested that at an equivalent concentration of Al, the particle size of Alhydrogel^®^ was reduced when formulated in PBS [95] relative to physiological saline [89]. Others have also postulated that antigenic adsorption onto AH in the presence of phosphate results in the formation of more compact particles, which in turn improves the efficacy of the vaccine [96]. Given the high adsorptive capacity of hydroxylated aluminium for inorganic phosphate, this first result is slightly perplexing. For example, Tang et al. proposed that while ionic exchange was the predominant means by which phosphate was adsorbed by highly crystalline boehmites, an additional mechanism was observed with regards to more amorphous derivatives, which involved the precipitation of aluminium phosphate [97]. Lookman et al. also supported this hypothesis, demonstrating that the precipitation of aluminium phosphate was restricted to the surface of aluminium hydroxide in systems exposed to high concentrations of phosphate [98]. Aluminium phosphate itself has been identified as an efficient chemical binding agent [99] and it is therefore more likely that its presence within these suspensions may serve as a nexus for particulate agglomeration in the absence of competing antigens.

#### The particle size of ABAs at the site of intramuscular injection post administration

The extracellular milieu found at the site of intramuscular injection is a diverse environment rich in anionic and proteinaceous ligands for aluminium. Some examples include phosphate [100], citrate [101] and serum components [100]. In situations where the association between antigen and adjuvant are limited, the former have been reported to contribute to significant levels of antigenic elution in vitro [102–104]. Such a scenario yields particulates whose surfaces are almost devoid of antigenic material providing a suitable site for protein adsorption in situ. This results in the formation of a hard corona whose speciation and magnitude shapes the biological identity and size of ABAs. Interstitial proteins are also known to compete for ABAs facilitating the displacement of antigen and subsequent evolution in protein corona composition [105].

The solubility of aluminium salts in biological fluid is largely dependent upon their individual physicochemical properties and is likely to be partially stymied due to the protective influence of this proteinaceous corona. However, smaller entities available for independent migration away from the injection site may, theoretically, still exist. It is reasonable to assume, therefore, that in response to injection related trauma, the increase in interstitial pressure will facilitate the rapid and independent removal of these species through the lymphatics [106], resulting in their presence within the draining lymph nodes [8] and bloodstream [107, 108] prior to leukocytic recruitment [109]. Furthermore, the translocation of these species is likely to be more pronounced for AH than AP at earlier junctures due to the more rapid initial dissolution rate of the former in vivo [107, 108]. It is therefore not unreasonable to suggest that the independent migration of such species may provide one of the initial stimuli for the immunological response potentiated by aluminium salts.

The remaining adjuvant material available at the injection site resides predominantly within the micron range and represents the greatest deposit of extracellular aluminium whose clearance and translocation will be dependent upon ingestion by APCs [110]. While the kinetics of internalisation will be different with regards to the dynamic environment found in vivo [109], uptake of particles by APCs is likely to occur relatively quickly after administration. The size of particles encountered within this timeframe will likely induce a predominantly phagocytic mechanism of uptake, although there will still be some discrete populations available for receptor-mediated endocytosis [111]. Materials composed of smaller particles are more avidly internalised by APCs [64, 70, 75, 112]. However, the requirement of ABA phagocytosis in order to potentiate an immune response has been questioned by several reports, which demonstrated an absence of material within the intracellular compartment of APCs despite the generation of robust adaptive immunity [29, 73, 113]. Such events appear counter-intuitive considering the essential role of phagocytosis in the destruction and direction of the immune response against pathogenic bacteria [114], the majority of which have sizes residing in the region of 1–4 μm [115]. In addition, cells of a monocytic lineage typically exhibit a highly conserved ruffled membrane [116], which has been implicated in the preferential uptake of particles whose sizes correspond to this range, although the attachment and subsequent internalisation of 2–3 μm structures is considered optimal [117]. Furthermore, the variation in abundance of particles existing below *ca* 3 μm provides a novel explanation concerning the relationship between particle size and the magnitude of adjuvant uptake by phagocytes observed in vitro. The application of this theory to ABAs reveals that materials containing a greater abundance of these particles upon exposure to biological medium are more readily endocytosed than their larger counterparts and are also able to target more of the surrounding cellular population (AH > AP) [64]. Larger particles were also observed within the intracellular environment of THP-1 cells, although the number of these entities was severely restricted. Such trends emphasise the importance of size with regards to the operation of the phagocytic pathway, which is often hindered by larger particles due to the relationship between the kinetics of cell signalling and manifestation of inhibitory signals [118]. Nevertheless, larger particles often have greater amounts of aluminium associated with their structure and the internalisation of a few of these entities may result in the delivery of a greater concentration of aluminium into the intracellular compartment of phagocytic cells. This may provide an initial justification for the observation that larger aluminium adjuvant particles promote greater levels of cytotoxicity in vitro [64].

### The role of zeta potential in the immunological response towards ABAs

There are a plethora of mechanisms by which antigens typically associate with the surface of ABAs. ABAs exhibit distinct differences in PZC permitting the adsorption of a specific spectrum of pathogenic and allergenic proteins at *ca* pH 7.4, which is equivalent to that experienced within the vaccine preparation and at the injection site [119, 120]. For example, AH, which is positively charged at physiological pH, will form electrostatic associations with negatively charged species such as BSA thereby facilitating surface adsorption. By contrast, AP, which is negatively charged within the afore-mentioned pH range, exhibits a higher affinity for positively charged antigens such as lysozyme. Although this model is well established, further studies revealed that while attractive forces including electrostatic interactions, hydrogen bonding, hydrophobic and van der Waal forces contributed to the absorption of proteins by aluminium salts, adsorption was not guaranteed in these systems if repulsive forces predominated in situ [121]. The recent immobilisation of AH upon a quartz crystal microbalance sensor also revealed that the adsorption of BSA by this material could not be adequately explained by electrostatic mechanisms alone [122]. In conjunction, these studies suggest that antigenic adsorption within vaccine preparations may occur through additional mechanisms and be more complex than originally contemplated.

Vaccine subunits exhibiting bipolar tendencies can also interact with both ABA compounds, although the orientation of antigen domain is inevitably dependent on the relative charges involved [123]. While variance in protein orientation appears to be redundant with regards to immunopotentiation in this case, structural degradation and unfolding [22, 124], expedited by the individual micro-environmental pH at the adjuvant surface [125], can promote enhanced antigen-adjuvant interaction [102, 126] followed by a concomitant decrease in antibody production [127]. However, this trend is not universally upheld for all such adjuvant-antigen complexes [128]. Phosphorylated antigens also target and preferentially bind hydroxylated surfaces through ligand exchange creating complexes with high associative strength [129].

The degree of antigenic adsorption to particulate entities is regarded as a crucial factor governing the potentiation of the immune response towards aluminium-adjuvanted vaccinations [130], facilitating T cell responses through improved cross presentation via enhanced cellular uptake [75, 131]. Indeed, when administered subcutaneously the elution rate of antigens adsorbed by electrostatic mechanisms was greater than those held by ligand exchange, consistent with the enhanced retention of the latter at the site of inoculation [60, 132]. While antigenic retention may be considered beneficial with regards to interaction with infiltrating inflammatory monocytic populations, these studies were the first to highlight that the strength of antigenic adsorption is in fact inversely proportional to the antibody response mounted in vivo. Preparations with the largest degree of phosphate modification have typically exhibited the lowest adsorptive affinities [60, 61, 94, 132] and thus potentiated the most substantial immune response in animal models [60, 61, 132]. Hence, phosphate treatment of AH has been successfully employed to improve the efficacy of vaccine formulations in several studies [61–63]. Furthermore, robust antibody responses are mounted against vaccines in which minimal adsorptive forces are displayed between antigen and ABAs [133, 134]. Despite the predominance of repulsive interactions, the latter observed an association between the two components in which the antigenic species became entrapped within adjuvant void spaces. This permitted simultaneous internalisation of antigen and adjuvant by APCs and provided a novel mechanism of immunopotentiation independent of antigen adsorption to aluminium salts.

While the zeta potential of aluminium salts influences the magnitude of proteinaceous adsorption observed within biological media, no correlation between this parameter and particulate uptake has been definitively demonstrated. The instantaneous nature of the adsorption of interstitial proteins by these adjuvants often results in the transformation of their surfaces to reflect negative charges of equivalent magnitude [64], making comparisons of this nature virtually impossible. Others have also struggled to demonstrate a significant role for surface charge with regards to both the efficacy of AH nanorod endocytosis and resultant titres of OVA specific IgG1 and IgE [135]. Previous studies investigating the coronal composition of cationic, neutral and anionic particles have demonstrated that the latter are more effectively opsonised through the association of IgG with their surfaces [136]. Furthermore, opsonised particulates of equivalent sizes exhibited greater levels of phagocytic uptake than those lacking this motif, an event which was mediated through interaction with Fc receptors on the surface of macrophages [137].

## Cellular uptake of aluminium adjuvants in human vaccinations

**Fig. 1:**
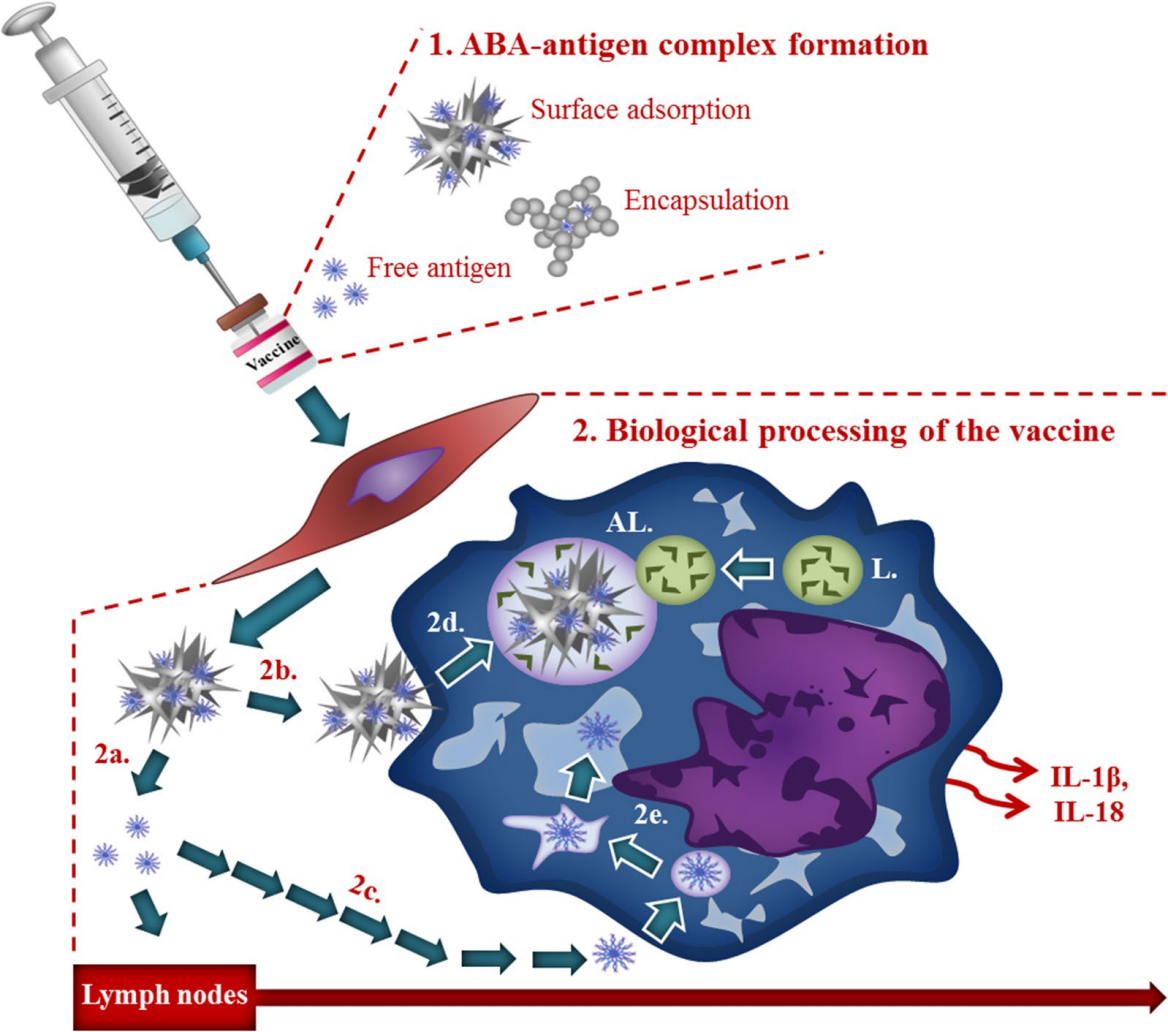
Biological processing of vaccines containing ABAs following intramuscular injection. 1. Vaccine preparation includes antigen adsorbed on the surface of the ABA, occluded within aggregates of ABA and, potentially, free antigen. 2. Biological fate of vaccine constituents includes; independent translocation of antigen (< *ca* 200 nm) to lymph nodes (2a); inflammatory monocyte/macrophage uptake of larger antigens through micropinocytosis (2c); intracellular antigens are either processed by the lysosomal system (L) or evade lysosomal capture through rupture of the endosome (2e); antigen associated with ABA (adsorbed or occluded) is internalised by phagocytosis (2b) and processed via autophagy, endosomes maturing into autophagosomes and ultimately, autolysosomes (AL) (2d). The biological mechanisms involved in the processing of these various entities contribute to the downstream activation of complexes such as the NALP-3 inflammasome and the subsequent release of pro-inflammatory cytokines including IL-1β

Advances in modern-day vaccine engineering have allowed for the production of highly purified protein antigens in large quantities that boast excellent safety profiles [138]. Unfortunately, immunisation using antigen only results in little to no antibody production. Therefore, it has remained necessary to include particulate substrates in vaccines to overcome this barrier [138, 139]. It is the immunostimulatory properties of ABAs, described as their ‘adjuvanticity’ that has driven their predominant and continued use in human vaccinations [138]. Of the fundamental mechanisms governing the adjuvanticity of ABAs are their ability to provide enhanced delivery of antigens to APCs, subsequently initiating an immune response and high antibody titers (Fig. 1) [10, 138]. For this reason, when ABAs are formulated in vaccines they are generally added in excess [138].

Some debate has ensued in the scientific literature as to the importance of the binding avidity of the ABA to the antigen in potentiating and shaping the resultant immune response [10, 60, 138]. If the antigen remains tightly adsorbed to its ABA as for studies using alpha-casein and Alhydrogel^®^ respectively, then such high-affinity interactions diminish the immunostimulatory properties of the vaccine [60]. As such, the choice of antigen in a vaccine dictates its ability to dissociate from its ABA [10], yet is often solely chosen dependent upon its charge. Weak electrostatic forces of attraction result in the rapid dissolution of the antigen in muscle interstitial fluid (MIF) at the injection site [10]. As such, it cannot be presumed that the entirety of ABA particulates remain co-adsorbed to their highly purified target antigen [6]. These effects have been described previously as the depot effect of ABAs, whereby the presence of aluminium in the vaccine prolongs the persistence of the antigen at the injection site [10, 139]. This theory has since been disputed as injection sites could be excised without affecting the adjuvanticity of the administered ABAs [140, 141].

Ligand and electrostatic interactions still give rise to antigen persistence at the injection site [109]. Therefore, two general modes of action for ABAs may be surmised in explaining their immunostimulatory effects in vivo. Firstly, increased antigen persistence by ABAs allows for sufficient recruitment and accumulation of infiltrating inflammatory cells to initiate an immune response at the injection site [10, 109]. Secondly, the subsequent association of the ABA to the antigen promotes their interaction and potential cellular uptake by infiltrating APCs, further driving the initiation of an immune response [109]. Thus, ABAs are able to potentiate the immune response whether co-adsorbed or dissociated from their target antigen in a vaccine.

### Cellular mechanisms of aluminium adjuvant uptake

The scientific literature to date has almost entirely focused upon the cellular uptake of the antigen in a vaccine formulation. Consequently, the question remains as to how the cellular uptake of ABAs and their transport to locations distant from the injection site contributes to this process [6, 10, 60, 109, 139]. In drawing comparisons between the cellular uptake of the two ABAs used most frequently in clinical vaccinations, Alhydrogel^®^ and Adju-Phos^®^, consideration must be given to the cellular routes of uptake of ABAs and the types of cells recruited to the site of injection. The process of cellular uptake is stringently controlled at the plasma membrane of the cell, which allows for both the passage of substances from the extracellular space into the cell cytosol via endocytosis and their expulsion from the intracellular space via exocytosis [142]. Therefore, the interchange between these two important cellular transport mechanisms will ultimately be responsible for driving the cellular passage of ABAs [142, 143].

#### Non-specific endocytic mechanisms of cellular uptake

Non-specific mechanisms of endocytosis can be defined as cellular uptake mechanisms whereby internalisation is achieved without the use of specific receptors such as protein transporters embedded in the cell membrane [142]. Upon the administration of the vaccine formulation to the injection site, the degree of heterogeneity with regards to the ABA and antigen species formed, inevitably increases [6]. Therefore, administration of an ABA into the extracellular space would likely drive its dissociation into non-particulate forms [144]. Therefore, immunisation with an ABA would be expected to release the free solvated trivalent metal cation, ${\text{Al}}^{ 3+ }_{{({\text{aq}})}}$, at the injection site [6]. As the principal bio-reactive species of aluminium, the release of ${\text{Al}}^{ 3+ }_{{({\text{aq}})}}$ into physiological milieu would have the potential in vivo for complexation, forming high and low molecular weight charged and neutral soluble species of the metal ion [144].

In addition, these ‘break-down’ products of ABAs may also be released into MIF once degraded intracellularly and exocytosed from cells and hence represent all forms of non-particulate ABA. Endocytic mechanisms are tightly-regulated by the size of the extracellular substance [142]. Therefore, all non-particulate forms of ABAs of a size greater than a few nanometres would not be expected to diffuse through cellular pores of sub-nanometre dimensions, but rather cross cellular membranes by non-receptor mediated endocytosis [142–144]. These cellular mechanisms of uptake may occur by simple diffusion known as transcellular transport, against an electrochemical gradient or require energy in the form of adenosine triphosphate (ATP) for uptake by active transport, cross ion channels or pores in the cell membrane or alternatively traverse the extracellular space through cell layers via paracellular transport [6, 142].

Paracellular transport is achieved through selectively permeable barriers that consist of epithelial and endothelial cell layers such as those found in the blood–brain barrier (BBB), forming tight junctions between adjoining cells [6]. In the presence of aluminium, residual leakiness of these membranes including the BBB has been noted, thereby diminishing the ability of tight junctions to restrict the passage of substances into the brain, promoting the free diffusion of aluminium between cells [144]. It remains unlikely however, that paracellular transport would allow for the free cellular passage of nano- or micron-sized particulates of either Alhydrogel^®^ or Adju-Phos^®^ due to the close proximity of even compromised cell layers [143]. Furthermore, as this cellular transport mechanism fails to result in the direct cellular uptake of at least particulate ABAs, other mechanisms of cellular internalisation would be expected to occur once particles interact with the cell membrane (Fig. 1) [142, 143].

Transcellular transport does not require specific receptors on the cell membrane, thereby acting as a diffusive process of cellular uptake. Since an unequivocal mechanism has yet to be elucidated for the transcellular transport of aluminium, combined with the expected blockade of this mechanism of cellular transport via the intracellular accumulation of ${\text{Al}}^{ 3+ }_{{({\text{aq}})}}$, little evidence supports a significant role for this route of uptake [144]. Low molecular weight neutral complexes of aluminium would, however, accumulate over time in cells via their transcellular diffusion through the plasma membrane and via ion channels into cell cytosol. Charged low molecular weight complexes of aluminium are predicted to be internalised via active transport [144], driven through variances in charge between exoplasmic vesicle membranes and the cytosolic cell face [145]. Finally, high molecular weight complexes of aluminium carrying neutral charge would be likely internalised in vesicles via non-specific adsorptive endocytosis [143, 144].

The amorphous nature of Adju-Phos^®^ as an AP based adjuvant would be expected to more readily dissociate to give ${\text{Al}}^{ 3+ }_{{({\text{aq}})}}$, than semi-crystalline Alhydrogel^®^, an AH based adjuvant. Therefore, while both ABAs would give rise to increased concentrations of the trivalent metal cation at the injection site, the more rapid dissociation of Adju-Phos^®^ would be predicted to result in a greater proportion of high and low molecular weight soluble complexes of aluminium [144]. As such, immunisation using Adju-Phos^®^ may result in the reduced loading of nanometre and micrometre-sized particulate aggregates versus Alhydrogel^®^, thus lowering the adsorptive capacity of the ABA to protein antigens [6, 140]. Therefore, in the absence of unequivocal data concerning these latter mechanisms of cellular uptake, a role for the adsorptive capacity of ABAs upon the potentiation of the immune response remains to be elucidated.

#### Endocytic mechanisms governing the cellular uptake of particulate aluminium adjuvants

All pathways of endocytosis result in the cellular internalisation of ABA particles, ultimately resulting in their presence within lysosomal compartments in cell cytosol [144]. Specialist variations of endocytosis can be defined under six recognised pathways consisting of phagocytosis, macropinocytosis, clathrin-mediated endocytosis, caveolar-mediated endocytosis, pinocytosis and adsorptive or non-receptor mediated endocytosis [142, 143]. Phagocytosis is a form of endocytosis and a process of cellular uptake that relies upon mechanical interactions of phagocytic cells with apoptotic or necrotic cells, cellular fragments, pathogens or particles that fall in the approximate size range of 0.5–5 μm and is only possible by a limited number of cell types [109, 117]. As an actin-regulated mechanism of engulfment, reorganisation of the actin cytoskeleton is necessary to allow for cellular uptake [144]. This mechanism of uptake can often be of detriment to the cell, however, due to the necessity of the formation of large pores in the cell membrane. As such, phagocytosed substances are engulfed through cup-shaped protrusions in the cell membrane that gradually approach and close around particulate matter [143].

Phagocytosis is one of the most recognised cellular pathway governing the internalisation of micron or nano-sized particulates of ABAs and is controlled by the molecular process of autophagy [144, 146]. The entry of particulate material into the cell initiates the formation of the phagophore of shape and size dictated by the engulfed substance [143]. The recruitment of the cytosolic light chain protein 3 (LC3) to the phagophore, forms a double-membrane known as an autophagosome which is vesicular in nature and encompasses engulfed particulates as well as mitochondria and peroxisomes [147]. Peroxisomes are found in all eukaryotic cells and are small membrane-bound cytoplasmic cellular organelles that contain a host of enzymes including catalase and urate oxidase that participate in a variety of oxidative mechanisms [145].

In the latter stages of autophagy and most notably for macroautophagy [147], a lysosome combines with the autophagosome triggering its maturation into an autolysosome, following the dissociation of LC3 from the membrane. The lysosomal contents spill into the autolysosome releasing enzymes including cathepsin B which acidify the resultant vesicular compartments to approximately pH 4.0 [146]. This results in the degradation of internalised particulates of the ABA, thereby releasing ${\text{Al}}^{ 3+ }_{{({\text{aq}})}}$ into cell cytosol [6, 144, 146]. The release of cathepsin B has been suggested to act as an endogenous danger signal that in combination with degradative products of ABAs, triggers localised inflammation at the injection site [148].

Phagocytosis is the optimal mechanism of cellular uptake when particles are in the size range of 1–3 μm (Fig. 1) [117, 142]. Macropinocytosis as a phagocytic cellular uptake mechanism would likely govern the cellular uptake of particulate material ≤ 1 μm owing to their maximal outreaching pore size of a few hundred nanometres in the extracellular space [142]. As with phagocytosis, this cellular process of uptake only occurs in a limited number of phagocytic cells and requires the rearrangement of the actin cytoskeleton, though arguably to a lesser extent to allow for the cellular passage of smaller sub-micron-sized particles [142, 143]. In this process, a large volume of extracellular fluid containing particulate matter is engulfed via the ruffling of the cell membrane that gives rise to varying shapes of the vesicles upon closure. Large cellular organelles known as macropinosomes [143, 149] then form, which may also account for the internalisation of lower molecular weight species of aluminium [144].

Clathrin-mediated endocytosis engulfs particles through the formation of cytoplasmic clathrin-coated pits that initiate a process of self-assembly into polygonal-shaped cages, facilitating cellular uptake [143]. This mechanism of uptake is more likely to play a role in the uptake of the free protein antigen in a vaccine [6], of which an adsorbed ABA would only be internalised if its size was less than that of clathrin-coated vesicles of approximately 100 nm [142, 143]. Caveolin-driven endocytosis results in the formation of caveolin protein-coated cytoplasmic hairpin-like depressions, up to 80 nm in diameter. While both Clathrin and caveolin-endocytic cellular mechanisms of particulate uptake are able to occur through receptor-mediated and independent mechanisms, their contributory role to the uptake of particulate ABAs is likely to be minimal. Finally, small sub-nanometre particles may enter the cell via simple diffusion across the cellular phospholipid membrane bilayer [143], as with lower molecular weight species of aluminium [144].

#### Intracellular tracing of protein antigens via fluorescent-labelling methodologies

Owing to the difficulties encountered in controlling the particle size of amorphous preparations of aluminium adjuvants, polystyrene particles have previously been used to assess the effects of particle size upon their ability to potentiate the immune response. As with the use of nanometre-sized ABA particles, greater immunopotentiation was noted for particles of 230 nm versus larger particles, following their intradermal injection in mice [150]. As with previous studies of this nature, however, the method relied upon the adsorption of OVA to adjuvant particles. OVA is frequently used as an antigen in vaccine research to study the effects that ABA elicit upon the immune response [73, 150]. The predominant reason for the use of this so-called ‘model antigen’ is the ease in which it may be conjugated to the fluorescent tag fluorescein isothiocyanate (FITC), subsequently allowing for its cellular internalisation to be conferred via flow cytometry, fluorescence and or confocal microscopy [73].

Green fluorescent protein (GFP) has also been used to allow for the cellular uptake of labelled protein antigens to be determined [151]. As these large fluorescent molecules may dissociate from their adsorbed antigen in both in vitro cell culture and in vivo animal models, the identification of these probes within cells cannot be used to infer the intracellular presence of the ABA. While intrinsic fluorescence of the protein antigen only in the ultraviolet range of the electromagnetic spectrum has allowed for its concentration to be determined when associated to its ABA, labelling of the vaccine would likely be necessitated to allow for its intracellular tracing [152]. Building upon these approaches, new methods have been devised to unequivocally identify ABAs, allowing their cellular fate to be determined in cellular models of vaccination [7, 153].

#### Non-phagocytic mechanisms of immunopotentiation for aluminium adjuvants

The Nalp3 (or NLRP3) inflammasome is an intracellular multi-protein complex containing a pattern recognition receptor (PRR) composed of leucine-rich repeats (LLRs) that belong to the nucleotide-binding oligomerisation domain (NOD)-like receptor family [37]. Upon assembly, this large intracellular protein machinery forms a complex with apoptosis-associated speck-like protein containing a caspase recruitment domain and caspase-1 (ASC) [37]. The activation of the inflammasome by particulate and or crystalline ABAs has been shown to promote the secretion of mature IL-1β and IL-18 that are cleaved from the pro-interleukins by activated caspase-1 that subsequently enhance the immune response [37]. Antibody production has however been noted, without activation of the Nalp3 inflammasome [154, 155].

A non-phagocytic pathway has been suggested for ABAs in their ability to enhance the delivery and cellular uptake of protein antigens by professional APCs, while evading cellular internalisation of the adjuvant [113]. Therein, the interaction of ABAs adsorbed to OVA protein antigens at the cell surface of activated DCs was found to trigger lipid sorting and in turn, the activated DC surface was found to initiate a non-phagocytic delivery mechanism of the antigen only [113]. Although such a process of abortive phagocytosis has been proposed for ABAs, in the same study the semi-quantitative analysis of DCs exposed to ABAs via transmission electron microscopy (TEM), occasionally revealed apparent electron-dense particulates internalised within the cell cytoplasm [113, 156]. Therefore, DCs are suggested to retain their phagocytosing capacity at the injection site and hence a role for the cellular uptake of ABAs upon the potentiation of an immune response cannot be overlooked. Furthermore, as crystalline ABAs have been suggested to act as damage-associated molecular patterns (DAMPs) and activators of the Nalp3 inflammasome, their cellular uptake may be contributory if not essential for the adjuvanticity of ABAs [37, 154].

#### A recognised cellular pathway of immunopotentiation by aluminium adjuvants

ABAs have been suggested to potentiate the immune response by providing an enhanced delivery mechanism of the co-adsorbed antigen to DCs [138]. ABAs are also thought to be able to increase the antigen uptake and antigen presentation on the cell surface of activated DCs via major histocompatibility complex class II molecules (MHCII) [151]. Therefore, the enhanced number of MHCII-presented antigen sites upon DCs are thought to be able to engage naïve CD4^+^ T cells that upon binding differentiate, dependent on the cytokines released by DCs, initiated by the transcriptional nuclear factor-κB (NF-κB) entering the nucleus of the DC [113, 138]. The subsequent activation of the Nalp3 inflammasome by particulate ABAs via the activation of the SyK and PI3K pathway produces IL-18 and IL-1β that are released into the extracellular matrix along with the pro-inflammatory IL-17 cytokine (Fig. 1) [37, 113]. The differentiated T lymphocytes then activate B cells that in turn produce an increased number of antibodies and predominantly that of IgG, thereby driving humoral-mediated immunity and a T_H_2 response [10, 156].

### Cell recruitment

ABAs have been found to recruit a host of innate immune cells to the injection site including neutrophils, eosinophils, immature DCs, monocytes, macrophages and natural killer cells [34, 109, 142, 143]. The effects that ABAs elicit upon the relative abundance of each aforementioned cell type, their migration from the injection site and their capacity to promote the cellular uptake and presentation of the antigen to adaptive immune cells, directly impacts upon their ability to potentiate the immune response [10, 37, 80, 109].

Immunofluorescence of excised injection sites from immunised mice using fluorescently-labelled antibodies has been used to identify infiltrating cell types [8]. It has been found that regardless of whether the ABA is administered via intraperitoneal or intramuscular injection [109, 142], neutrophils are found to dominate the injection site only a few hours following vaccination [34]. As a phagocytic cell line, the arrival of neutrophils at the injection site would likely result in the engulfment of particulates in the size range of 0.5–5 μm [142], although phagocytosis of the ABAs would be more pronounced following the arrival of monocytes after 24 h and the vast recruitment of macrophages following 7 days [109].

A marked increase in the presence of eosinophils are typically observed at day 7 following immunisation, concomitant with increased numbers of infiltrating MHCII-positive (+) DCs at the injection site [35, 109]. In spite of their non-phagocytic nature, eosinophils have been found to be contributory to the promotion of humoral-mediated immunity and hence the production of antibodies in the presence of ABAs [109]. Monocytes are able to differentiate into both macrophagic and dendritic cell types and increased numbers of MHCII^+^ DCs at the injection site at day 7, have been linked to their presence [34, 109]. Overall, the rapid infiltration of innate immune cells is thought to be critical for the initiation of an adaptive immune response and the production of antibodies of which MHCII^+^ DCs have been found imperative for the adjuvanticity of ABAs and the activation of the Nalp3 inflammasome [37, 113, 156].

### Research methods for the identification of intracellular aluminium adjuvants

Lumogallion [4-chloro-3-(2,4-dihydroxyphenylazo)-2-hydroxybenzene-1-sulphonic acid] is a fluorescent stain for aluminium and has been used successfully for both the quantitative and qualitative determination of aluminium in natural waters [157] and biota [158], respectively. Lumogallion was originally used for the fluorimetric detection of aluminium in sea and river water owing to its extremely low limit of detection of 2 nM comparable to inductively coupled plasma mass spectroscopy (ICP-MS) [159]. The proposed 1:1 coordination of aluminium to the fluorophore is suggested to occur via the two phenolic oxygen ions and the azo group binding to the metal ion, forming complex rings in aromatic linkages. In this manner, lumogallion has been proposed to act as a planar tridentate ligand for aluminium [159].

To date, the fluorescent stain morin [2′,3,4′,5,7-Pentahydroxyflavone] has remained the most frequently used fluorophore for the detection of aluminium in plant and animal tissues producing a green fluorescence emission at 420 nm, through coordinating 3:1 with the metal ion [160]. Unlike morin, lumogallion produces no detectable fluorescence in the presence of divalent metal cations including physiologically relevant concentrations of calcium and magnesium [159, 161]. Furthermore, while lumogallion is known to bind to iron, this results in the formation of a non-fluorescent complex that upon excitation at 500 nm achieves no detectable fluorescence maxima at *ca* 590 nm (orange), unlike that observed in the presence of aluminium [161]. Therefore, lumogallion has high specificity and selectivity for the binding and detection of aluminium, respectively. Such properties have rendered the fluorophore invaluable for the unequivocal identification of aluminium in both cells [7, 65, 144, 153, 162, 163] and tissues [164–166].

With regards to ABAs, lumogallion has allowed for the unequivocal identification of AH-based adjuvant formulations, including Alhydrogel^®^ within monocytic THP-1 cells [7]. As an immunologically relevant cell line [64] monocytes are known to be one of the first phagocytosing cell lines recruited to the site of vaccination and hence act as a significant carrier or vehicle of particulate ABAs, away from the injection site [109]. Using fluorescence microscopy complemented with TEM, vesicle or endosome-like structures were identified in cell cytosol of approximately 0.5–1 μm in diameter [7]. While it might be expected for a phagocytosing cell line to internalise micron-sized adjuvant particles into lysosomal compartments, all such published data prior to this study provided little or equivocal data of such. Therefore, this study highlighted lumogallion as an excellent fluorescent molecular probe for the identification of particulate ABAs [7, 153].

The use of lumogallion as a sensitive fluorescent molecular probe for aluminium [7] has revealed the unequivocal identification of particulates of Alhydrogel^®^ and Adju-Phos^®^ in vesicle-like structures in the cytoplasm of a relevant monocytic THP-1 cell line [64]. For both adjuvant formulations those particulates internalised were of approximately 1 μm in size and were found exclusively in the cell cytoplasm. As Alhydrogel^®^ was found internalised at every concentration of the adjuvant, this demonstrated its greater cytoplasmic loading versus Adju-Phos^®^ whereby uptake was diminished at 50 and 100 μg/mL of the ABA. As such, this may infer that AH based adjuvants are more prone to migrate from the injection site [64]. The release of extracellular DNA from dying cells has been found to enhance the adjuvanticity of ABAs, as DAMPs that activate the Nalp3 inflammasome [138, 167]. Therefore, the reduced cellular uptake of Adju-Phos^®^ and its enhanced cytotoxicity at the injection site in comparison to Alhydrogel^®^ may act to mediate its adjuvanticity by an alternative cellular pathway of immunopotentiation [64].

### Direct fluorescent labelling of vaccine antigens

Attempts to monitor the cellular uptake of antigens in vaccine formulations have frequently made use of commercially available fluorochromes or conjugated immunolabels for detection via fluorescence [168] and electron microscopy [169], respectively. While such labels are readily detectable under the respective imaging modalities [170], whether or not they remain co-localised to their target antigen is less well-established. Such limitations are further compounded when tracing vaccine antigens in complex physiological environments, mimicking those conditions encountered in vivo. Furthermore, the inevitable alterations to the physiochemical properties of the antigen and its co-adsorbed antigen would likely affect the mechanisms by which their cellular uptake subsequently ensues [7, 64, 89].

Recently, the use of direct-fluorescent labelling has allowed for the conformation and intracellular fate of a model amyloid-β_42_ (Aβ_42_) peptide antigen to be followed in a cellular-based model of vaccination [163]. Therein, simulated vaccines were prepared adjuvanted with lumogallion-labelled ABAs that were subsequently co-cultured with THP-1 cells. The longer amino acid variant of amyloid, Aβ_42_, has frequently been implemented in the aetiology of Alzheimer’s disease, depositing in brain tissue as extracellular senile plaques, in a β-sheet rich conformation [171–173]. These aberrant misfolded deposits are suggested to break down into neurotoxic oligomeric Aβ species, triggering a progressive cascade of deleterious effects [171]. Thioflavin T (ThT) binds to mature amyloid fibrils in a β-pleated sheet conformation that thereby allowed for the detection and intracellular fate of the antigen to be assessed [163].

This new approach to directly label the moiety of interest suggests that Aβ_42_ is endocytosed by THP-1 cells through micropinocytosis [174] of which its co-formulated ABA (Alhydrogel^®^, Adju-Phos^®^ or Imject^®^ Alum) is processed via the alternative pathway of autophagy into late-stage autolysosomes [163]. The ability of Aβ to evade or disrupt lysosomal capture has recently been implicated in separate studies in both monocytic [175] and neuronal cell lines [168, 174]. The amyloidogenic protein alpha (α)-synuclein that forms intraneuronal inclusion or Lewy bodies in Parkinson’s disease have also been found to disrupt the formation of autolysosomes [176]. Therefore, the ability of amyloidogenic proteins to evade lysosomal capture may explain their intracellular persistence in vivo [163, 168, 174–176]. Furthermore, controlled lysosomal rupture has been shown to activate the NALP-3 inflammasome and hence such mechanistic modes of action may underlie the immunogenicity of vaccine components [177, 178]. Taken collectively, these studies highlight the importance of monitoring the cellular fate of both the antigen and adjuvant constituents of a vaccine in understanding the resultant immune response in vivo [163]. In conclusion, these approaches may greatly benefit current and future clinical trials utilising immunotherapy for the eradication of neurodegenerative diseases and other complex disease states [179].

## Conclusion

Human exposure to aluminium is burgeoning [180] with significant implications for human health [166, 181]. ABAs are effective and cheap but are they safe? Confirmation of their safety remains to be addressed and will only come from further research on their biological activities at injection sites and beyond. All ABAs currently in use in vaccination and sub-cutaneous immunotherapy require further validation of their safety, which means that the manufacturer of AAHS should release this ABA so that its safety can be verified independently. Burgeoning knowledge regarding the biological activities of ABAs now dictates that their safety should be evaluated independently of their presence in vaccine formulations.

## Abbreviations

AAHS: amorphous aluminium hydroxyphosphate sulphate
Aβ: amyloid-β
ABA: aluminium based adjuvant
AH: aluminium oxyhydroxide
AP: aluminium hydroxyphosphate
APC: antigen presenting cell
ASC: apoptosis-associated speck-like protein
ATP: adenosine triphosphate
BBB: blood brain barrier
BSA: bovine serum albumin
CD4^+^: cluster of differentiation-4 positive
CD8^+^: cluster of differentiation-8 positive
DAMPs: damage associated molecular patterns
DAPI: 4′,6-diamidino-2-phenylindole, dihydrochloride
DC: dendritic cell
DLS: dynamic light scattering
Fc: fragment crystallisable (antibody) region
FITC: fluorescein isothiocyanate
GFP: green fluorescent protein
HBsAg: hepatitis B surface antigen
HLA: human leukocyte antigen
HPV: human papilloma virus
ICP-MS: inductively coupled plasma mass spectroscopy
IgE: immunoglobulin E
IgG: immunoglobulin G
IL: interleukin
IR: infrared spectroscopy
ITAM: immunoreceptor tyrosine activation motif
LC3: light chain protein 3
LRR: leucine rich repeat
Lumogallion: 4-chloro-3-(2,4-dihydroxyphenylazo)-2-hydroxybenzene-1-sulphonic acid
MHCII: major histocompatibility complex class II
MIF: muscle interstitial fluid
Morin: 2′,3,4′,5,7-pentahydroxyflavone
Nalp3: neuronal apoptosis inhibitory protein, leucine rich repeat and pyrin domains-containing protein 3
NF-κB: nuclear factor-κB
NOD: nucleotide-binding oligomerisation domain
OVA: ovalbumin
PBMC: peripheral blood mononuclear cells
PBS: phosphate buffered saline
PCB: poorly crystalline boehmite
PI3K: phosphoinositide-3-kinase
PLA: polylactide
PRR: pattern recognition receptor
PSD: particle size distribution
PZC: point of zero charge
SyK: spleen tyrosine kinase
TEM: transmission electron microscopy
THP-1: T helper 1
T_H_2: T helper 2
ThT: Thioflavin T
UPW: ultrapure water
XRD: X-ray diffraction
